# Determination of Selected Metals in Fruit Wines by Spectroscopic Techniques

**DOI:** 10.1155/2017/5283917

**Published:** 2017-10-31

**Authors:** Justyna Płotka-Wasylka, Małgorzata Rutkowska, Bartłomiej Cieślik, Alan Tyburcy, Jacek Namieśnik

**Affiliations:** Department of Analytical Chemistry, Faculty of Chemistry, Gdańsk University of Technology, 11/12 Narutowicza Street, 80-233 Gdańsk, Poland

## Abstract

**Background:**

The determination of metals in different types of food and beverages samples has drawn significant attention due to several reasons with the most important one being the nutritional and toxic effects of these elements or their compounds. The knowledge of certain elements content in wines/fruit wines is of special interest due to their toxicity in case of excessive intake and also the effect they seem to have on the organoleptic properties of wine.

**Results:**

The study was focused on measuring the concentration levels of trace metals in fruit wines. Analysis of K, Ca, Fe, Zn, Cd, Mg, Pb, Sn, and Hg in so-called* homemade fruit wine* was carried out by AES, AAS, CV-AAS, and GF-AAS techniques. The calculated calibration curves showed good linearity range for all tested analytes (with coefficient of determination in the range from 0.989 to 0.999). The low values of the limit of detection (from 0.0031 *μ*g/L to 0.47 mg/L) and the limit of quantification (from 0.009 *μ*g/L to 1.41 mg/L) were obtained.

**Conclusions:**

The allowed levels of metal in fruit wines are prescribed by the International Office for Grapes and Wines (OIV). The data obtained from the study area for all metals did not exceed the international limits.

## 1. Introduction

The determination of appropriate metals in different type of biological, environmental, and food samples has drawn significant attention due to several reasons with the most important one being the nutritional and toxic effects of these elements or their compounds [1]. Very important is also knowledge of content of various metals in alcoholic drinks including regional and homemade wines and fruit wines. Without any doubt, wine is widely consumed beverage in the world with very obvious commercial value and social importance. Moreover, there is many wineries that produced fruits wine (alcoholic beverage made from variety of base ingredients, other than grapes), however, in many countries so-called homemade fruit wines still dominate on table at important family ceremonies. A big variety of fruits which differ in shape, taste, colour, and nutritive value are available in the market and many are utilized widely for production of fermented beverages.

Numerous researches have been conducted to present the fact that the moderate consumption of wine (particularly red) improves good health and longevity when it is combined with a balanced diet [2]. Scientific discussions related to human exposure to the trace metals contents of dietary products and various beverages, including wines and fruit wines, have received growing attention, since the consumption of these drinks, especially reasonably large volumes, may significantly contribute to the daily dietary intake of trace elements such as K, Ca, Mg, Cr, Co, Fe, F, I, Cu, Mn, Mo, Ni, Se, and Zn by humans [3, 4]. In addition, some of these trace elements such as Cu, Fe, and Mn cause an organoleptic effect and also contribute to the haze and taste of wines. Contrarily, several metals and metalloids, such as Cd, Pb, Sn, Hg, and As, are known to be potentially toxic. At the same time, the analysis for certain elements in wines and fruit wines is of special interest due to their toxicity in case of excessive intake and also the effect they seem to have on the organoleptic properties of these alcoholic beverages. A typical example is copper which is an essential as well as a potentially toxic element for humans when in excess [5]. In contrast, the excessive presence of the elements like Al, Cu, Fe, and Zn has a definite negative effect on the organoleptic properties of the different kinds of wine. Moreover, the content of some metals can be used for the identification of the area where the wine comes from [5]. Information on metals mainly occurred in wine and fruit wine are given in Table 1.

It is well known that the presence of these elements in wine and fruit wine can influence the wine making process or can change the taste and quality of the final product [1]. Trace metals are commonly present in these alcoholic drinks usually coming from the two main sources: environmental, that is, soil on which grapevine, fruit, or herb are grown and contamination originating from cars, factories, and so forth, and anthropogenic, including use of fertilizers and pesticides and oenological practices (machinery, piping, use of fining agents, additives, etc.) [6].

Taking into account the above-mentioned information, the monitoring of metals (both essential and potentially toxic) content in wine and fruit wine is of great importance mainly due to control of quality and authenticity of these alcoholic drinks, metals' bioavailability, and toxicity. Several publications on investigation of metals concentration in fermented alcoholic drinks exist. Among the reported studies wine samples are not rare. Different methods of metal analysis were employed in these studies with the majority being atomic absorption and atomic emission [6]. The following methods have been reported for studies in relation to atomic absorption techniques: Flame Atomic Absorption Spectrometry (F-AAS) [7–10], Electrothermal Atomic Absorption Spectrometry (ET-AAS) [5, 11], Hydride Generated Atomic Absorption Spectrometry (HG-AAS) [12, 13], and Cold Vapour Atomic Absorption Spectrometry (CV-AAS). Moreover, studies dealing with the methods in relation to atomic emission techniques have also been reported. To these methods the following can be included: Inductively Coupled Plasma-Optical Emission Spectrometry (ICP-OES) and Inductively Coupled Plasma- Mass Spectrometry (ICP-MS) [14–18]. Alongside these other rare metal analysis techniques like spectrophotometric analysis, X-Ray Fluorescence (XRF), and Near IR Spectroscopy have been reported [19–21]. The majority of the studies are focused mostly on Italian and Spanish wines. Information on determination metals content in Argentinian, Spanish, Romanian, Turkish, and Croatian wines can also be found (absorption techniques: Flame Atomic Absorption Spectrometry (F-AAS)) [8, 12, 14, 15]. All of these publications are dealing with wines and fruit wines produced on an industrial scale while there is lack of information on metals content in regional and homemade fermented alcoholic drinks including fruit wines. Moreover, no reports exist on the metals determination in such rare beverages which are fruit wines produced in Poland.

In this study, 17 fruit wine samples of so-called homemade fruit wine made from different kinds of fruits were taken for analysis. A method of quantitative analysis for the determination of selected metals (K, Ca, Fe, Zn, Cd, Mg, Pb, Hg, and Sn) in these fruit wines by AES, AAS, CV-AAS, and GF-AAS dependent on the analysed metal was validated and applied. The choice of analytes was based on a literature review. These metals were selected which were pointed in literature to be the most commonly present in wine. Moreover, metals such as mercury, cadmium, and lead enter the environment primarily as a consequence of industrial emissions or via disposal of products containing these metals, including mercury-cadmium or cadmium-nickel batteries. This type of industry is located near the fruit growing area of one of the fruits from which the fruit wine was produced.

To the best of our knowledge, there is no data concerning the metal analysis in so-called homemade fruit wine samples; therefore, this work brings new knowledge in this field. Due to the fact that both regional and homemade products are becoming more popular, it is significant to monitor this kind of products to protect human life and health.

## 2. Materials and Methods

### 2.1. Materials

For mineralization a mixture of oxidizing agents and acids was used: nitric acid, 65%, Suprapur grade supplied by Merck company, and hydrochloric acid, 36%, Suprapur grade supplied by Merck company. Standards used for calibration solution preparations were as follows:

- Ca standard for AES, 10000 mg/L, supplied by BWB Technologies UK Limited
- Cd standard solution for AAS, 1000 ± 4 mg/L in 2% HNO_3_, supplied by Fluka
- Fe standard, 1000 mg/L in 2% HNO_3_, plasma grade supplied by SPEX CertiPrep
- K standard for AES, 10000 mg/L, supplied by BWB Technologies UK Limited
- Mg standard solution for AAS, 1001 ± 6 mg/L in 2% HNO_3_, supplied by Fluka
- Pb standard solution for AAS, 1000 ± 4 mg/L in 2% HNO_3_, supplied by Fluka
- Zn standard, 1000 mg/L in 2% HNO_3_, plasma grade supplied by SPEX CertiPrep
- Hg standard-MSHG for CV-AAS, 100.48 ± 0.22 *μ*g/mL in 3.3% HCl purchased from Inorganic Ventures, Inc. (USA)
- Sn standard solution for AAS, 1000 ± 4 mg/L in 2% HNO_3_, supplied by Fluka.

Firstly standard solution with intermediate concentration of 10 mg/L was prepared by diluting 1000 mg/L stock solution for all determined elements. For Flame Atomic Absorption Spectrometry (F-AAS) and Atomic Emission Analysis (AES) measurements, series of calibration solutions with proper concentrations were made. For Graphite Furnace Atomic Absorption Spectrometry (GF-AAS) measurement, one basic standard solution was prepared for every element. Concentrations of calibrations were as follows:

- Ca: 10; 20; 30; 40; 50 mg/L for AES analysis
- Cd: 0,1; 0,3; 0,5; 0,7; 1,0 mg/L for F-AAS analysis
- Cd: 0,002 mg/L for GF-AAS analysis
- Fe: 0,5; 1,0; 1,5; 2,0; 2,5 mg/L for F-AAS analysis
- K: 10; 20; 30; 40; 50; 60 mg/L for AES analysis
- Mg: 0,4; 0,6; 0,8; 1,0; 1,2; 1,5 mg/L for F-AAS analysis
- Pb: 0,5; 1,0; 1,5; 2,0; 2,5 mg/L for F-AAS analysis
- Pb: 0,05 mg/L for GF-AAS analysis
- Sn: 0,1 mg/L for GF-AAS analysis
- Zn: 0,1; 0,3; 0,6; 0,8; 1,0; 1,2; 1,5 mg/L for F-AAS analysis.

Mercury standard solution for CV-AAS measurement was prepared as follows.

10 mg of L-cysteine (noncrystalline) was weighed and transferred into a 1000 mL volumetric flask. 2 mL of guaranteed-reagent grade concentrated nitric acid was added, shaken, and then filled up to the mark with deionized water to prepare 0.001% L-cysteine solution. The reagent was stored in a cool and dark place.

1 mL of 100 mg L^−1^ mercury solution was placed in a 100 mL volumetric flask and diluted up to the mark with L-cysteine solution to obtain 1 mg L^−1^ Hg.

For GF-AAS analysis proper modifiers were used:

- Phosphate modifier for graphite furnace AAS, NH_4_H_2_PO_4_  100 ± 2 g/L in H_2_O supplied by Merck company for Sn, Ni, and Cd analysis
- Magnesium nitrate-palladium nitrate matrix modifier 0,2% Mg and 0,3% Pd in 1% HNO_3_ supplied by MS Spektrum for Pb analysis.

### 2.2. Equipment Used

For samples mineralization Multiwave GO digestion system supplied by Anton Paar company was used. For Ca and K analysis Flame Photometer BWB-1 supplied by BWB Technologies UK Limited was used. For moderate concentration heavy metals determination, Flame Atomic Absorption Spectrometer SensAA supplied by GBC Scientific equipment Pvt. Ltd. (Australia) with dual beam optical system and air acetyl flame was used. Deuterium lamp for background correction and hollow-cathode lamps as radiation source were installed. For low concentration heavy metals determination, Graphite Furnace Atomic Absorption Spectrometer Savant AAZ supplied by GBC Scientific equipment Pvt. ltd (Australia) with Zeeman background correction was used. As a carrier gas technical grade argon was supplied and hollow-cathode lamps were installed as radiation source. Mercury/MA-2000 supplied by Nippon Instruments Corporation (NIC, Japan) was used to analyse mercury by cold vapour technique and purified dry air was used as the carrier gas.

### 2.3. Sampling

A total of 17 samples of so-called homemade fruit wine made from different kinds of fruits were obtained from people who produced these drinks for themselves. All the samples were stored at room temperature (21°C) protected from light. Information on analysed fruit wine samples is given in Table 2.

### 2.4. Preparation of the Fruit Wine Samples for Analysis

**Figure 1:**
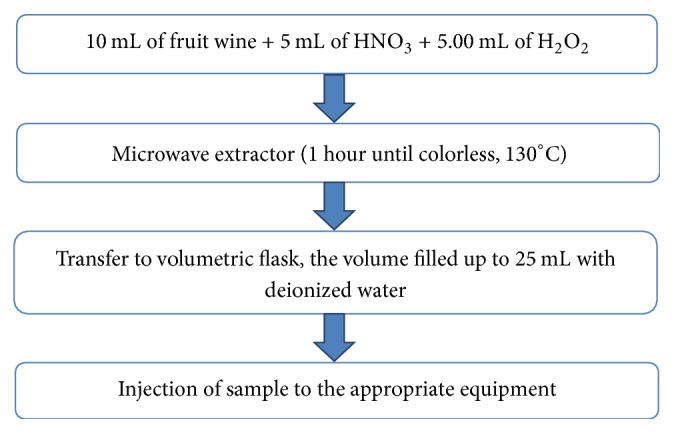
Procedure of sample preparation for spectroscopic analysis.

The procedure of the sample preparation is presented in Figure 1. The fruit wine samples were treated with hot HNO_3_–H_2_O_2_ for decomposition of organic matrix. Two different samples were taken from each fruit wine and therefore, after separate digestion, two different solutions were obtained for each sample all of which were analysed three times with appropriate equipment. The original solutions of extracts were 1 : 1 diluted to measure the Ca, Fe, Pb, Zn, and Cd content, 1 : 20 diluted to measure the Mg content (1 : 25 for sample number 10), and 1 : 10 diluted to measure K content. The original fruit wine samples with two types of additives (additive B: activated alumina were obtained from Nacalai Tesque, Inc., Kyoto, and Wako pure Chemical Industries, Ltd. (Japan); additive M: sodium carbonate + calcium hydroxide) were inserted to the appropriate device to measure Hg content.

### 2.5. Measurements Conditions

In case of AES analysis, K and Ca were determined jointly. In case of AAS analysis, subsequent measurement was carried out one element at a time using proper hollow-cathode lamp for the specific wavelength. The wavelength used for Cd, Fe, Mg, Pb, Zn, Sn, and Hg analysis was, respectively, 228,8 nm; 248,3 nm; 285,2 nm; 217,0 nm; 213,9 nm, 235,5 nm; 253.7 nm. The linear regression method was used for the calibration curve. For the GF-AAS analysis proper furnace temperature programs were used. The measurement conditions for GF-AAS analysis are described in Table 3.

### 2.6. Quality Assurance

The calibration of the measuring instrument was performed using one of the techniques of the external calibration, the calibration curve method using the appropriately prepared standard solutions of metal ions tested. Working calibration standard solutions were prepared by diluting standard stock solutions containing each of target compounds in the appropriate amounts of deionized water. Linear range for analytes of interest was studied by replicate analysis of the standard stock solutions. The linear regression values were calculated with the average absorbance of three replicate injections for each analyte. The calculated calibration curves showed good linearity range for all tested analytes. The linear regression for each analyte with coefficient of determination in the range from 0.989 (Hg) to 0.999 (Mg) is presented in Table 4. Coefficient of variation (CV) was the average value of different concentrations of examined compounds in the linear range and was in the range from 1.5% (Mg) to 10% (Hg), which is considered good method precision. Sensitivity of the developed method was considered in terms of limit of detection (LOD). Limit of detection (LOD) and limit of the quantification (LOQ) of the methods have been set according to OIV recommended technique [22]. The two limits were based on values of the standard deviation of the intercept (Sa) and they were deduced of mathematical expressions: LOD = (3,3*∗*Sa)/*b* and LOQ = 3*∗*LOD. The obtained results are presented in Table 4.

### 2.7. The Uncertainty of Measurement

The expanded uncertainty of measurement is a quantity defining an interval about the result of a measurement that may be expected to encompass a large fraction of the distribution of values that could reasonably be attributed to the measurand. The dominant parameter taken into consideration during the development of procedures was repeatability of measurements.

The value of the expanded uncertainty of measurement was calculated by using

(1) $$\begin{array}{c} U=k\times \frac{SD}{\sqrt{n}}, \end{array}$$

where

  SD is standard deviation of measurements;
 
*n* is number of measurements;
 
*k* is coverage factor (*k* = 2, defines an interval having a level of confidence of approximately 95%).

On the other hand, the calculated value of the confidence interval for the series of results is described by

(2) $$\begin{array}{c} \Delta x=t\left(\;\alpha ,f\;\right)\frac{SD}{\sqrt{n}}. \end{array}$$

For the 0.05 significance level and the number of degrees of freedom *f* → *∞*, a critical parameter Student's* t*-test is approximately 2.

## 3. Results and Discussion

Due to the fact that the samples were digested in the mixture of HNO_3_ and H_2_O_2_, the presence of an organic matrix is improbable. However, some ions which may cause interference like HPO_4_^ ^^2−^, H_2_PO_4_^ ^^−^, H_3_P_4  _, and Cl^−^ exist but are at very low level of concentration. The existence of SO_4_^ ^^2−^ ions in wines is obvious and several researches were conducted to investigate the possible relationships between the content of sulfate and selected metals [23]. Information on determined concentration of selected metals in analysed samples is presented in Table 5. The results reveal the amounts of Cd metals to be at trace level (0.423 ± 0.027 to 18.4 ± 1.3 *μ*g/L). The content of Pb, Zn, and Fe metals is also very low (2.11 ± 0.23 to 116.3 ± 5.3 *μ*g/L, 11.8 ± 0.21 to 316 ± 6.8 *μ*g/L, and 0.423 ± 0.027 to 0.969 ± 0.052 mg/L, resp.). In some cases, Cd, Pb, and Fe concentrations remained below the limit of detection and could not be detected (iron was not determined in 10 samples). The results show that the contents of Mg and Ca are at a similar concentration level (5.00 ± 0.34 to 29.7 ± 1.9 mg/L and 4.29 ± 0.42 to 50.1 ± 2.5, resp.). The results reveal the K content to be higher than the other elements in question (165 ± 11 to 441 ± 38 mg/L). The mean value of such elements as Zn, Fe, Mg, and Ca shows that they can be determined with the F-AAS method. On the other hand the Cd and Pb concentrations were far too low to be determined by the F-AAS method and were determined by using GF-AAS. Due to the fact that content of K was very high it could be detected by photometer. The content of toxic Hg in most samples was at ultra-trace level (below the limit of detection). Only in one of the samples was it possible to determine the total mercury content with the CV-AAS method (0.437 ± 0.026 *μ*g/L). The content of Sn in each of thesamples was under limit of detection.

Comparison of the relative abundances of the essential elements in white fruit wines reveals the following tendency: K > Mg ≥ Ca > Fe > Pb > Zn > Cd > Hg, Sn.

These relations are very similar in red and rose fruit wines: K > Ca > Mg > Fe > Zn > Pb > Cd > Hg, Sn.

This ranking is not to be taken as exact because there are many variables in analysed fruit wine production, mainly kind of fruits used in wine production, region, and climate. Therefore, the amount of an element may be found in a wide variety of ranges for different fruit wines even if they were produced from the same fruits.

Although the homemade fruit wine produced from different fruits is taken into consideration and therefore it is hard to compare the content of selected metals in these samples, the following general conclusions can be made from data in Table 4:

- Cd and Pb content of white wines are higher than that of red and rose fruit wines.
- Zn content of rose fruit wines is higher than those of white and red fruit wines.
- K, Ca, and Fe contents are at similar level in each of fruit wine.
- Mg content in white fruit wines is the same or very close to those of red fruit wines; in rose fruit wines the range of Mg content is very wide.
- Red, white, and rose fruit wine samples which were analysed did not contain toxic heavy metals such as Hg and Sn.

Maximum permissible level of selected metals in fruit wines does not exist; however, many countries have set maximum permissible level of some metals in wines considering both the enological and toxicological effects of the metals in wine. The allowed levels of metal in wines are also prescribed by the International Office for Grapes and Wines (OIV). The maximum permissible levels of selected metals in some countries and by OIV are given in Table 6 [4, 24].

Here, the estimated data (Table 5) demonstrate that the contents of all the metals in fruit wine samples are considerably smaller than the maximum concentrations allowed according to the OIV (Table 6). In addition, the determined concentrations of metals are much below the permissible concentrations, which is probably due to the fact that the analysed fruit wines are made from different kinds of fruits except grapes. Moreover, the contents of these metals in the studied homemade fruit wines were mostly significantly lower than in some European and American wines (Table 7). Comparison of the concentration of metals in so-called homemade fruit wines produced in Poland analysed during this study and published data on wines of different origin is presented in Table 7.

The K levels in homemade fruit wines were in range of 165–441 mg/L. These wines had similar interval to that reported for French wines. The homemade fruit wines had lower levels of K compared to the Czech, German, Greek, Hungarian, Italian, Spanish, and American wines. The calcium concentration range in homemade fruit wines was 4.29–50.1 mg/L and is comparable to that in Greek wines but much lower than the Czech, French, German, Hungarian, Italian, Spanish, and American wines. The magnesium levels in the homemade fruit wines were in the range of 5.0–29.7 mg/L and are much lower than that reported for each country. The Pb levels in the homemade wines were in the range 2.11–116.3 *μ*g/L. These levels are similar to that reported for Spanish wines and much lower than that reported for wines produced in other countries. The Zn levels in analysed in this study wines are very poor in zinc and the concentration is much lower than in wine coming from other countries. The comparison made for Cd levels in the homemade fruit wines and wines of different origins showed that the wines analysed in this study are among the intermediate levels. The Cd levels in Ethiopian wines are similar to that reported in Spanish wines, higher than in Czech, French, and Italian wines but lower than in Greek and Hungarian wines. The iron concentration in wines depends on several factors, mostly the soil; however iron levels may increase in wines due to the usage of steel devices during production. Iron is of importance to the wine maker because when it is present at >7–10 mg/L, it may cause cloudiness or colour change; the content depends upon iron levels in soil and dust and contamination during harvesting, transportation, and processing [24]. The Fe contents in the homemade wines were in the range 0.423–0.969 mg/L and are much lower than that reported for wines produced in other countries. This is probably due to the fact that homemade fruit wines are mainly produced in carboy, not in steel devices.

## 4. Conclusions

Nowadays, an important course of action should be the monitoring of regional products that in large numbers are going to the consumers. Despite the fact that in Poland there are laws regulating the trade of the domestic alcohol product, it does not indicate the acceptable exposure limit of selected metals in such beverages. Moreover, huge amounts of wine and fruit wine from Polish wineries are going to the people, and no literature data on these products are available.

In this study a method of quantitative analysis for the determination of selected metals (K, Ca, Fe, Zn, Cd, Mg, Pb, Hg, and Sn) in fruit wines by AES, AAS, CV-AAS, and GF-AAS dependent on the analysed metal was validated and applied. The calibration of the measuring instrument was performed using the calibration curve method. The calculated calibration curves showed good linearity range for all tested analytes. LOD and LOQ of the methods have been set according to OIV recommended technique. The determination of concentration of selected metals in fruit wine samples was carried out. The contents of all the metals in these samples were considerably smaller than the maximum concentrations allowed according to the OIV. In addition, the determined concentration of metals is much below the permissible concentrations, which is probably due to the fact that the analysed fruit wine is made from different kinds of fruits except grapes.

Because the amount of published data on heavy metal content in wine samples is very limited and, in fact, there are no data on the heavy metal content in wine samples coming from regional products, these results can bring significant knowledge in this area.

## Figures and Tables

**Table 1:** Information on metals mainly occurring in wine [1–6].

Metal	Content, origin	Effects
Potassium	A natural component of grape. Its concentrations in wine reflect the levels in grapevine in the final stages of berry ripening.	High K levels affect the stability of wine with respect to the potassium hydrogen L-(+)-tartrate precipitation.

Calcium	A natural component of wine. The concentration of calcium in wine can be affected by the traditional practices of deacidification (CaCO_3_ addition) or plastering (CaSO_4_ addition).	Elevated calcium levels can lead in some wines to calcium L-(+)-tartrate precipitation.

Aluminum	It is found in grape juice, but the concentration in both juice and wine is elevated because of the use of bentonite and to a lesser extent from contact with aluminum surfaces.	It has become apparent that aluminum is strongly complexed in wine which affects its bioavailability from one side and makes haze formation unlikely from the other side.

Iron	It can be present at significant concentration in the juice of grapes, either through general environmental contamination (air borne dusts), or due to the application of fungicides in the vineyard. The reported Fe concentrations in juice range from 0.7 to 23.0 mg/L, with the highest concentrations from older studies when contamination from cast iron equipment was more common.	Above trace levels, iron plays roles: altering redox system of the wine in favor of oxidation, participating in the formation of complexes with tannins and phosphates thus resulting in instabilities. At low concentration *iron *plays an important role in metabolism and fermentation processes as an enzyme activator, solubilizer, and functional component of proteins.

Copper	It can be present at significant concentration in the juice of grapes, either through general environmental contamination (air borne dusts), or due to the application of fungicides in the vineyard. During fermentation a large proportion of Cu is precipitated with yeast cells and its concentration is thus reduced significantly.	In trace amounts it is an important inorganic catalyst for metabolic activities of microorganisms. At high levels it plays an important role in catalyzing oxidation of wine polyphenols.

Lead	Its concentration significantly increase in open-top vessels, in holding bins, and during pressing. Juice and wine stored in concrete or waxed wood have significantly higher concentration of lead compared to juice and wine stored in stainless steel. Moreover fining with bentonite or filtering with diatomaceous earth contributes further to final Pb concentration, while fermentation, both primary and secondary, removed Pb.	Its effects on people are disastrous even in very small quantities. Lead can be accumulated in biological systems becoming potential contaminants mainly along the alimentary chain. Symptoms of lead poisoning may include abdominal pain, constipation, headaches, irritability, memory problems, inability to have children, and tingling in the hands and feet.

**Table 2:** Information on analysed samples of so-called home-made fruit wine. To determine alcoholic content of wine Alcolyzer Wine M/ME (Anton Paar) was used.

Sample number	Type of wine	Main ingredient	Production year	Alcohol content
1	White	Apple	2013	16%
2	Red	Black lilac	2014	14%
3	Red	Chokeberry	2012	12%
4	White	Apple	2010	14%
5	White	Apple	2015	14%
6	Rose	Plum	2008	12%
7	Red	Black currant & mint	2008	12%
8	Red	Chokeberry	2015	14%
9	Rose	Red currant	2015	13%
10	Rose	Raspberry	2015	13%
11	Rose	Strawberry	2013	15%
12	Rose	Red currant	2013	14%
13	Rose	Plum & wild rose & quince	2011	14%
14	Rose	Red currant & mint	2008	11%
15	Red	Black currant	2007	13%
16	White	Quince	2005	12%
17	Rose	Strawberry	2014	16%

**Table 3:** Measurement conditions for GF-AAS analysis.

Element	Measurement conditions	Step	Final temperature [°C]	Ramp time [sec.]	Hold time [sec]	Carrier gas on/off
Pb	Sample volume: 10 [*µ*l]	1 Inject	50	1,0	2,0	on
		2	90	10,0	15,0	on
	Modifier volume: 5 [*µ*l] NH_4_H_2_PO_4_	3	120	15,0	10,0	on
		4 Ashing	700	10,0	5,0	on
		5	700	0,0	1,0	off
	Magnetic field: 1,10 [Tesla]	6 Read	2500	0,7	0,6	off
		7	2500	1,0	1,0	on

Cd	Sample volume: 10 [*µ*l]	1 Inject	50	1,0	2,0	on
		2	90	10,0	15,0	on
	Modifier volume: 5 [*µ*l] Mg(NO_3_)_2_ Pd(NO_3_)_2_	3	120	15,0	10,0	on
		4 Ashing	850	10,0	5,0	on
		5	850	0,0	1,0	off
	Magnetic field: 1,00 [Tesla]	6 Read	2100	0,7	1,0	off
		7	2100	1,0	2,0	on

Sn	Sample volume: 10 [*µ*l]	1 Inject	40	1,0	2,0	on
		2	90	10,0	15,0	on
	Modifier volume: 5 [*µ*l] Mg(NO_3_)_2_ Pd(NO_3_)_2_	3	125	15,0	10,0	on
		4 Ashing	800	10,0	5,0	on
		5	800	0,0	1,0	off
	Magnetic field: 1,00 [Tesla]	6 Read	2600	0,9	1,0	off
		7	2600	1,0	2,0	on

**Table 4:** Basic validation parameters obtained for each analyte by using developed method (*n*, number of standards in three replicates; *R*^2^, coefficient of determination).

Analyte	*n*	Equation	*R* ^2^	LOD	LOQ	Linearity range	CV [%]
K	6	*y* = 654.68*x* + 1733.1	0.998	0.47 mg/L	1.41 mg/L	1.41–60 mg/L	2.6
Ca	5	*y* = 26.053*x* + 285.27	0.994	0.415 mg/L	1.245 mg/L	1.245–50 mg/L	2.4
Mg	5	*y* = 0.3746*x* + 0.0107	0.999	0.021 mg/L	0.063 mg/L	0.063–1.200 mg/L	1.5
Pb	5	*y* = 0.0207*x* − 0.0041	0.992	0.0031 *µ*g/L	0.009 *µ*g/L	0.0093–2.5000 *µ*g/L	2.0
Zn	7	*y* = 0.1208*x* + 0.0023	0.998	0.027 *µ*g/L	0.081 *µ*g/L	0.081–1.500 *µ*g/L	1.9
Cd	5	*y* = 0.2805*x* + 0.0242	0.997	0.0087 *µ*g/L	0.026 *µ*g/L	0.026–2 *µ*g/L	3.1
Fe	5	*y* = 0.0271*x* − 0.0059	0.991	0.009 mg/L	0.027 mg/L	0.027–5 mg/L	1.7
Hg	5	*y* = 0.0112*x* + 0.0045	0.989	0.012 *µ*g/L	0.036 *µ*g/L	0.036–0.8 *µ*g/L	10.0
Sn	5	*y* = 0,0028*x* + 0,0101	0.990	9.9 *µ*g/L	32.5 *µ*g/L	32.5–100 *µ*g/L	n.d

**Table 5:** Information on determined concentration of selected metals in fruit wine samples.

Sample number	K [mg/L]	Ca [mg/L]	Mg [mg/L]	Pb [*µ*g/L]	Zn [*µ*g/L]	Cd [*µ*g/L]	Fe [mg/L]	Hg [*µ*g/L]	Sn [*µ*g/L]
1	330 ± 33	17.9 ± 1.3	19.0 ± 1.4	88.5 ± 4.9	86.9 ± 2.3	3.72 ± 0.38	0.432 ± 0.032	<0.036	<32.5
2	255 ± 21	4.29 ± 0.42	18.6 ± 1.1	95.2 ± 5.2	103 ± 4.9	18.4 ± 1.3	<0.027	<0.036	<32.5
3	165 ± 11	30.9 ± 1.9	18.0 ± 1.0	35.6 ± 2.1	276 ± 6.1	0.509 ± 0.043	0.508 ± 0.038	<0.036	<32.5
4	353 ± 29	28.1 ± 1.7	13.03 ± 0.91	116.3 ± 5.3	105 ± 1.7	1.11 ± 0.11	<0.027	<0.036	<32.5
5	233 ± 18	18.8 ± 1.1	19.2 ± 1.5	75.3 ± 4.4	36.1 ± 0.32	<0.026	0.508 ± 0.037	<0.036	<32.5
6	296 ± 19	20.7 ± 1.5	5.00 ± 0.34	9.91 ± 0.95	164 ± 3.2	<0.026	<0.027	<0.036	<32.5
7	441 ± 38	27.0 ± 1.8	13.05 ± 0.89	43.5 ± 2.5	99.1 ± 3.4	0.795 ± 0.045	<0.027	<0.036	<32.5
8	264 ± 14	50.1 ± 2.5	19.8 ± 1.5	7.21 ± 0.68	36.1 ± 0.35	0.578 ± 0.034	<0.027	<0.036	<32.5
9	238 ± 13	32.4 ± 1.9	7.99 ± 0.68	<0.009	70.7 ± 0.49	<0.026	<0.027	<0.036	<32.5
10	259 ± 14	24.5 ± 1.6	29.7 ± 1.9	2.11 ± 0.23	170 ± 6.3	<0.026	<0.027	<0.036	<32.5
11	369 ± 29	29.3 ± 1.6	22.1 ± 1.6	29.4 ± 1.9	80.8 ± 3.1	0.924 ± 0.047	<0.027	<0.036	<32.5
12	411 ± 35	23.7 ± 1.5	11.97 ± 0.87	8.98 ± 0.87	146 ± 5.1	<0.026	0.407 ± 0.028	<0.036	<32.5
13	233 ± 16	35.0 ± 2.0	10.58 ± 0.77	6.62 ± 0.56	132 ± 4.6	<0.026	0.432 ± 0.031	<0.036	<32.5
14	398 ± 38	9.11 ± 0.59	9.88 ± 0.69	<0.009	316 ± 6.8	<0.026	0.969 ± 0.052	<0.036	<32.5
15	254 ± 21	22.9 ± 1.8	15.8 ± 1.1	20.9 ± 1.8	11.8 ± 0.21	<0.026	<0.027	0.437 ± 0.026	<32.5
16	208 ± 14	25.7 ± 1.7	16.1 ± 1.3	8.51 ± 0.85	268 ± 7.3	0.667 ± 0.036	0.558 ± 0.040	<0.036	<32.5
17	402 ± 35	22.9 ± 1.5	11.86 ± 0.78	10.98 ± 0.91	138 ± 3.9	0.423 ± 0.027	<0.027	<0.036	<32.5

Data are means ± SD. ULOD: under the limit of detection. All analyses were repeated 6 times (2 subsamples of each wine in triplicate).

**Table 6:** The accepted limits of the metals content (mg/L) in wine in different countries and given by OIV.

Country	Concentration of metals (mg/L)						
	Al	As	Cd	Cu	Pb	Ti	Zn
Australia	—	0.10	0.05	5.00	0.20	—	5.00
Germany	8.00	0.10	0.01	5.00	0.30	1.00	5.00
Italy	—	—	—	10.00	0.30	—	5.00
Poland	—	0.20	0.03	—	0.30	—	—
OIV	—	0.20	0.01	1.00	0.15	—	5.00

**Table 7:** Comparison of the concentration of metals in wines of different origin [24].

Metal	Concentration of metals (mg/L) in different wine origin								
	Czech	French	German	Greek	Hungarian	Italian	Spanish	American	Poland
K	493–3056	265–426	480–1860	955–2089	489–1512	750–1500	338–2032	462–1147	165–441
Ca	40–100	65–161	58–200	14.0–47.5	51–164	30–151	12–241	17–94	4.3–50
Mg	7.8–138	55–96	56–105	82.5–122.5	72–174	53–115	50–236	100–245	5.0–29.7
Pb	0.010–1.253	0.006–0.023	—	ND–0.62	—	0.01–0.35	0.001–0.096	—	0.002–0.095
Zn	—	0.44–0.74	0.3–1.5	0.05–8.9	0.6–1.9	0.135–4.8	ND–4.63	0.75–3.60	0.036–0.316
Cd	0.000055–0.0033	ND–0.0002	—	ND–0.03	0.00014–0.54	0.0012–0.0016	ND–0.019	—	0.0005–0.0184
Fe	0.9–5.2	0.81–2.51	0.4–4.2	0.7–7.3	2.03–23.7	1.35–27.8	0.4–17.4	1.2–6.6	0.407–0.969
